# Experimental Study on Consolidation-Creep Behavior of Subgrade Modified Soil in Seasonally Frozen Areas

**DOI:** 10.3390/ma14185138

**Published:** 2021-09-07

**Authors:** Fuyu Wang, Weichen Pang, Ziqi Li, Haibin Wei, Leilei Han

**Affiliations:** 1 School of Transportation, Jilin University, Changchun 130022, China; wfy@jlu.edu.cn (F.W.); pangwc19@mails.jlu.edu.cn (W.P.); 2 WISDRI City Construction Engineering & Research Incorporation Ltd., Wuhan 430000, China; 35022@wisdriudc.com

**Keywords:** seasonally frozen area, subgrade modified soil, consolidation-creep behavior, experimental study, road engineering

## Abstract

Frost heaving and boiling are the most common road disorders due to the special climatic conditions in a seasonal frozen area. From the perspective of controlling road disorders in seasonally frozen areas and making effective use of industrial waste residue, two kinds of subgrade modified soil—crumb rubber modified fly ash soil (CRFS) and oil shale waste residue modified fly ash soil (OSFS)—were proposed by the research group. The research results proved that the two new subgrade fillers both have excellent engineering characteristics in cold areas, such as high strength and low thermal conductivity, and both have the function of waste utilization, giving them broad application prospects. In road engineering, the instability of slopes and retaining walls and the uneven settlement of the subgrade are closely related to soil creep, which are problems that cannot be ignored in road design and use. As a new material to treat road disorders in seasonally frozen areas, more attention should be paid to the continuous deformation property of modified soil under long-term load. The study on the creep characteristics of the modified soil can provide reliable parameters for the design of the modified soil subgrade and predict the settlement of the subgrade after construction, which is of great significance to the stability of the subgrade. In this paper, an experimental study on the consolidation–creep characteristics of two kinds of subgrade modified soil in a seasonal frozen region was carried out, the relationship between modified soil deformation and time is discussed, and the effects of different moisture contents and compaction degrees on the creep characteristics of modified soil were analyzed. The test results provide parameters for the engineering design of modified soil subgrade and provide data support for the popularization and application of modified soil in seasonally frozen subgrade.

## 1. Introduction

Within the territory of China, the permafrost area accounts for 21.5% and the seasonal permafrost area accounts for 53.5% of the territory. Due to the special climatic conditions in the seasonal frozen region, frost heaving and boiling occur frequently, which are the most common road disorders in Northeast, North, and Northwest China [1,2,3].

Silty clay widely distributed in Northeast China is subject to frost heaving, which needs to be improved to meet the requirements of a subgrade filling. There are many studies about crumb rubber and fly ash modified road materials [4,5,6,7]. The results show that modified soil has good mechanical properties. In particular, silty clay was improved and the application of rubber particles and oil shale residues in road engineering was studied by our research group [8,9,10,11]. The results of these studies proved the feasibility of using the two modified soils in road engineering. Both of the two modified soils have excellent engineering characteristics in cold areas, such as high strength and low thermal conductivity, as well as having an environmental protection function. They have good application prospects in subgrade filling in seasonal frozen areas. However, there has been little research on the consolidation-creep behavior of modified soils.

The creep of soil refers to the process whereby soil deformation develops slowly with time under constant stress. In road engineering, the instability of slopes and retaining walls and the uneven settlement of roadbeds are all closely related to soil creep. If the creep property of soil is ignored in the design and construction, great safety risks will often become apparent after the actual project is implemented.

The theory of soil rheology and consolidation explain the objective law of soil deformation from different perspectives, but in fact both reflect the timeliness of soil deformation [12]. Consolidation characteristics and creep characteristics of soil complement each other, and the stability and deformation of building foundations are closely related to the coupling effect of the two, while the study of the coupling effect of the two has important guiding significance for engineering practice.

Taylor and Merchant took the lead in considering the effect of creep in the consolidation analysis of soil and proposed the Merchant Model to simulate the deformation of the soil skeleton. Ho et al. [13] derived an exact analytical solution of the one-dimensional consolidated flow equation in unsaturated soil by using eigenfunction expansion and Laplace transform techniques. Chen et al. [14] studied the consolidation and seepage of laterally confined clay and pointed out that the secondary time effect was mainly generated by the action of shear stress. They proposed a new consolidation model assuming that clay was a Maxwell material and solved the differential equation of a one-way case. Zhou et al. [15] proved that the fractional EVP (elastic–viscoplastic) creep model is obviously better than the traditional integer EVP model. Tan et al. [16] used the Bayesian probabilistic method to identify all unknown parameters based on the consolidation data during the entire consolidation process and quantified their uncertainty through the obtained posterior probability density functions. Additionally, they also determined the optimal model from among nine models. Yu et al. [17] combined the elliptic-parabolic double yield surface model with the modified Komamura–Huang model, and proposed a new viscoelasto–viscoplastic model, and a finite element consolidation analysis method that can fully consider the influence of the construction process. The method of combining the creep model with the geotechnical model has been a pioneer in the application of creep-consolidation theory in engineering. Since then, a large number of studies have been carried out on the consolidation creep characteristics of soil.

Wang et al. [18] presented a semi-analytical method to analyze the creep and thermal consolidation behaviors of layered saturated clays due to surface loads. Wong et al. [19] proposed a methodology to decouple the creep-deformation component from the total deformation measured in oedometer tests, which can determine the intrinsic properties of a 1D normally consolidated curve and creep behavior. Wang et al. [20] proved that the constitutive model of backfill consolidation and creep can be used to describe the creep behavior of the backfill under the high stress conditions of confinement. Yin et al. [21] presented a new simplified hypothesis B method for calculation of consolidation settlement of a clayey soil with creep. Bezvolev et al. [22] presented a method for the correct determination of creep and nonlinear viscosity parameters during compression–consolidation testing. Rezania et al. [23] reproduced soft soil behavior, both after pile installation and subsequent consolidation, by using an advanced critical-state-based constitutive model, and used a time-dependent extension of the model to study soil creep and the significance of its consideration in the overall pile-installation effects. Ran et al. [24] established a coupling model of seepage, deformation, and settlement on the basis of fluid-solid coupling mechanics theory, so as to calculate the land subsidence. This model includes both primary consolidation and secondary consolidation. Chen et al. [25] systematically studying the coupling mechanism of a geotechnical stress–strain model and consolidation and rheology, established the nonlinear viscoelastic consolidation model, and verified the rationality of the model through calculation and analysis of actual engineering. Yu et al. [26] adopted an improved Merchant model to compile the finite element calculation program of the nonlinear viscoelastic consolidation model and carried out consolidation-creep finite element analysis on the settlement of a soft soil foundation. Tang et al. [27] established a nonlinear viscoelastic consolidation creep model, in which the Duncan–Chang model was used to describe the instantaneous elasticity of soil and the Burgers model was used to describe the viscoelasticity. Sun et al. [28] established a new one-dimensional consolidation differential equation based on the Terzaghi one-dimensional consolidation theory with the modified Singh–Mitchell empirical creep model. Liu et al. [29] analyzed the influence of the consolidation and creep characteristics of the composite soil on the stability of a dam foundation, established an improved nonlinear Nishikan model, and obtained the analytical formula of viscoplastic strain rate. The vertical displacement distribution and reinforcement range of the dam structure under various working conditions were obtained through an example.

As for tests about consolidation and creep behavior, Jia et al. [30] conducted a series of triaxial unloading tests with frozen clay subjected to long-term high-pressure *K*_0_ consolidation before freezing, which indicated that the creep behavior of frozen clay is affected by the consolidation time and consolidation stress. Ghezal et al. [31] used chemical admixtures and commercially ternary blended cements to produce self-consolidating concrete, which showed that the magnitude of flexural creep varies widely depending on the properties of the polycarboxylic chemical admixture in use. Kamoun et al. [32] carried out disturbed clay samples to analyze their creep behavior, which showed that the variation of axial creep strain is correlated with time in a semi-logarithmic function. Jarad et al. [33] investigated the impact of temperature on the consolidation behavior of saturated compacted clays, which showed that the pre-consolidation pressure of both clays decreases as the temperature increases, while it decreases as the strain rate decreases, and the creep index increases as the temperature increases for both clays. Ghio et al. [34] studied the compressibility and creep of a diatomaceous soil from Mejillones Bay in northern Chile, which indicated that undisturbed samples are over-consolidated, although highly compressible after yielding and have significant creep strains. Rezania et al. [35] studied the one-dimensional (1D) time-dependent behavior of natural and reconstituted London Clay samples under saturated and unsaturated conditions. The tests results revealed stress dependency and suction dependency of primary and secondary consolidation responses of the soil samples. Ter-Martirosyan et al. [36] proposed that identification of concrete moisture content depending on relative air humidity, the groundwater filtration flow, and the influence of the filter cake at the contact between ground and concrete, make it possible to take account of the effect of soil conditions on concrete creep. Wang et al. [37] studied the influence of water content and normal stress on the coupling characteristics of consolidation and creep of loess, deduced the stress–strain time relationship equation of loess, and verified the model by using BP neural network. Zhang et al. [38] observed the samples of soft soil in the Huangshi area before and after creep by scanning electron microscopy, analyzed its consolidation and creep characteristics and microscopic mechanism, and concluded that improving the drainage property of soil in the process of soft soil foundation reinforcement can increase its consolidation degree and reduce the influence caused by creep.

Based on the research status of consolidation and creep of soil mentioned above, it has been found that most of the research on soil creep characteristics has been focused on soft soil and clay, and the applicable empirical model and theoretical model have been established respectively. As a new material to treat road diseases in seasonally frozen areas, more attention should be paid to the continuous deformation property of modified soil under long-term load. For this paper, an experimental study on the consolidation and creep characteristics of two new types of subgrade modified soil was carried out, the relationship between the deformation of the modified soil and time is discussed, and the influence of water content and compaction degree on the creep characteristics of the modified soil was analyzed. Some meaningful conclusions were obtained, which can provide reliable parameters for the design of modified soil subgrade and guide practical engineering applications.

## 2. Materials and Methods

### 2.1. Raw Materials

The crumb rubber particles used in this paper were taken from Changchun Rubber Products Factory and are recycled products of waste rubber products. Although the crumb rubber particles could produce certain harmful substances and cause pollution, such pollution is within the prescribed range [9,11,39]. The particles are reliable with uniform size and no impurities, of diameter 1–1.5 mm, and apparent density 1.29 g/cm^3^.

The oil shale waste residue used in this paper was semi-coke residue, produced in Wangqing County, Jilin Province, with an apparent density of 2.4 g/cm^3^ and a specific surface area of 16.2 m^2^/g.

The fly ash used in this paper was taken from a thermal power plant in Changchun City, which is Grade F, Class I, Si-Al type fly ash. When dry, it is a fine powder and has the nature of volcanic ash.

The silty clay used in this paper was taken from the homogeneous soil layer below 10 m of a construction site in Changchun City, and is used for typical subgrade filling in Northeast China.

Based on the research results in references [10,39], the optimal mix ratio of the two modified soils is given. The mass percentage of the crumb rubber modified fly ash soil (CRFS) is as follows: fly ash: silty clay: rubber particles = 32.7:65.3:2; the mass percentage of the oil shale waste residue modified fly ash soil (OSFS) is as follows: oil shale waste residue: fly ash: silty clay = 2:1:2.

According to the Specifications for Design of Highway Subgrades (JTG D30-2015) [40], the specific indexes of CRFS and OSFS are shown in Table 1.

### 2.2. Composition Analysis

**Figure 1:**
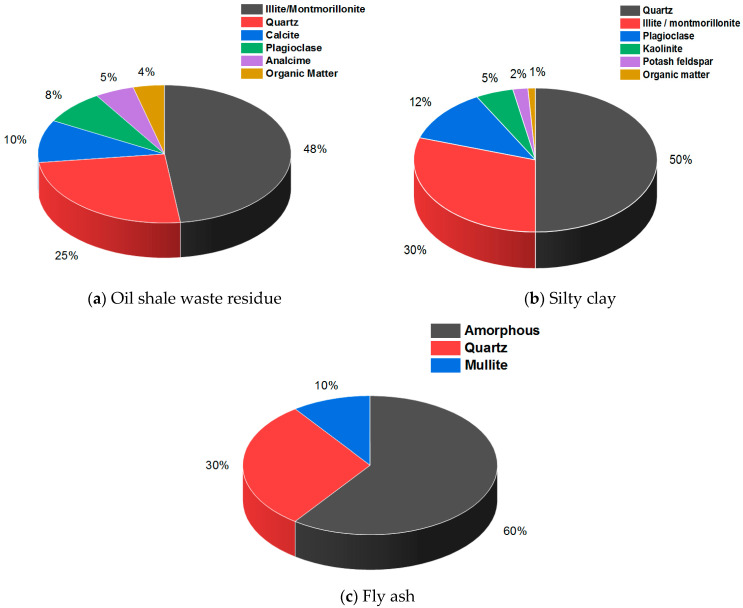
The mineral composition of the three raw materials.

The mineral and chemical constituents of oil shale waste residue, fly ash, and silty clay were analyzed by X-ray diffraction (XRD) and Fourier transform infrared absorption spectroscopy (FTIR), which are shown in Figure 1 and Table 2.

As shown in Figure 1 and Table 2, oil shale residue and silty clay are composed of quartz, illite, and montmorillonite, while fly ash is composed of quartz, mullite, and amorphous substance. The chemical composition of the three raw materials is similar, and they all contain a lot of SiO_2_ and Al_2_O_3_.

### 2.3. Preparation of the Modified Soil

**Figure 2:**
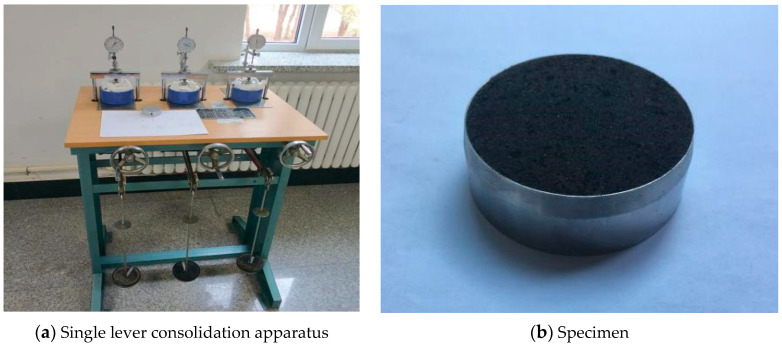
Diagrams of test apparatus and specimen.

One-dimensional consolidation-creep test was carried out in this study. The test apparatus is a single lever consolidation apparatus. The sample is a cylindrical soil column with a diameter of 61.8 mm and a height of 20 mm, as shown in Figure 2.

The sample was prepared by the static pressure method. All kinds of raw materials are pretreated by crushing and drying to prepare uniform modified soil with different moisture content, and the processed material is sealed for 24 h to ensure sufficient moisture diffusion. According to the compaction degree, the quality of the modified soil required by the ring cutter specimen is calculated, and the soil is pressed into the ring cutter by the static pressure method. Finally, the surface of the ring cutter is scraped flat with the soil scraper. The prepared sample is wrapped with plastic wrap to prevent moisture loss for subsequent tests.

### 2.4. Test Scheme

One-dimensional consolidation-creep tests were carried out for CRFS and OSFS respectively. The test process is as follows:

1. Sample preparation. Soil samples were prepared according to the Test Methods of Soils for Highway Engineering (JTG E40-2007) [41], with 5 samples for each of the two modified soils, as shown in Table 3.
2. Sample installation. The sample is loaded into the protective ring and placed on the pervious stone. The top surface of the sample is placed on the pervious stone and the pressure transmission plate and placed in the middle of the pressure frame. Directions are as follows. Turn the hand wheel clockwise until the lever reaches the top, then turn it counterclockwise 1 or 2 times. Make the pressure head face to face with the pressure plate and adjust the screws on the beam so that the container can be freely taken and put. Before starting the test, apply a 1 kPa preload (25.5 g) and adjust the dial gauge to zero. After preloading, the load can be applied step by step.
3. Loading scheme. The loading method adopted in this paper is hierarchical loading, and the instrument leverage ratio is 1:12. The loading sequence is 25-50-100-200-400-800 kPa, that is, the loading ratio is 1. During the experiment, wet cotton was used to surround the upper and lower permeable water surfaces to avoid moisture evaporation. The reading time points are 0 s, 15 s, 1 min, 2 min, 4 min, 6 min, 9 min, 12 min, 16 min, 20 min, 25 min, 35 min, 45 min, 60 min, 90 min, 2 h, 4 h, 10 h, 23 h, 24 h at the beginning of the test. The data were then recorded every 24 h. When the deformation of the specimen is less than 0.005 mm/d, that is, the deformation in two days is less than 0.01 mm, it can be considered that the deformation is stable, and the next level of load can be applied.
4. Test data processing and analysis.

## 3. Results and Discussion

### 3.1. Strain–Time Curve Analysis

**Figure 3:**
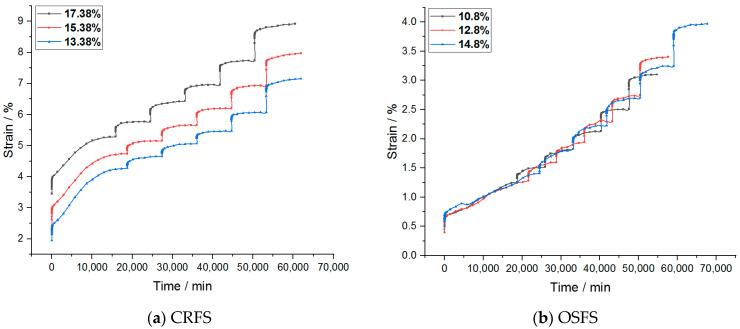
Strain–time curves of samples with different water contents.

**Figure 4:**
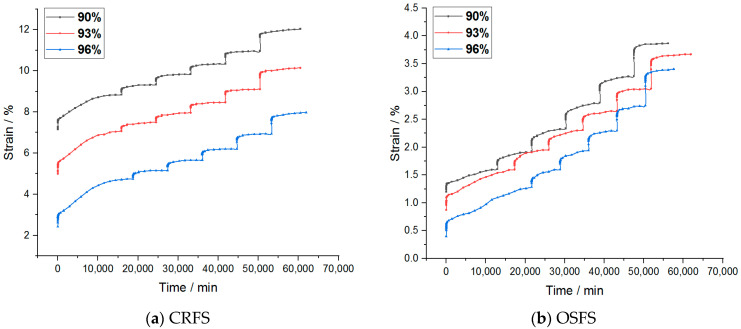
Strain–time curves of samples with different compaction degrees.

The strain–time variation curves of the two modified soils under different water contents and compactness are shown in Figure 3 and Figure 4.

It can be seen from the figures that the creep deformation of OSFS is smaller than that of CRFS, which is about 40% of the latter. It is also less affected by water content and compaction degree. The strain–time curves of the sample in the first few load stages almost coincide. The possible reason is that the OSFS contains less fly ash and less pores between the soil particles.

It is to be noted that the strain–time curve of the first load stage is abnormal. The possible reason is that when the load is applied, the gas discharge rate is much faster than the pore water discharge rate because the soil sample is not completely saturated. The reduction of gas volume accounts for a large part of the deformation of the soil samples, so the initial deformation rate is faster. With the discharge of gas, the soil deformation in the subsequent stages is mainly caused by the discharge of pore water, so the deformation rate decreases gradually. Therefore, the first stage of load is omitted in the subsequent analysis.

**Figure 5:**
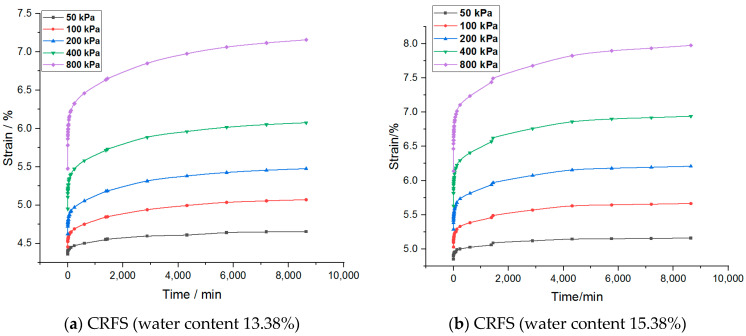

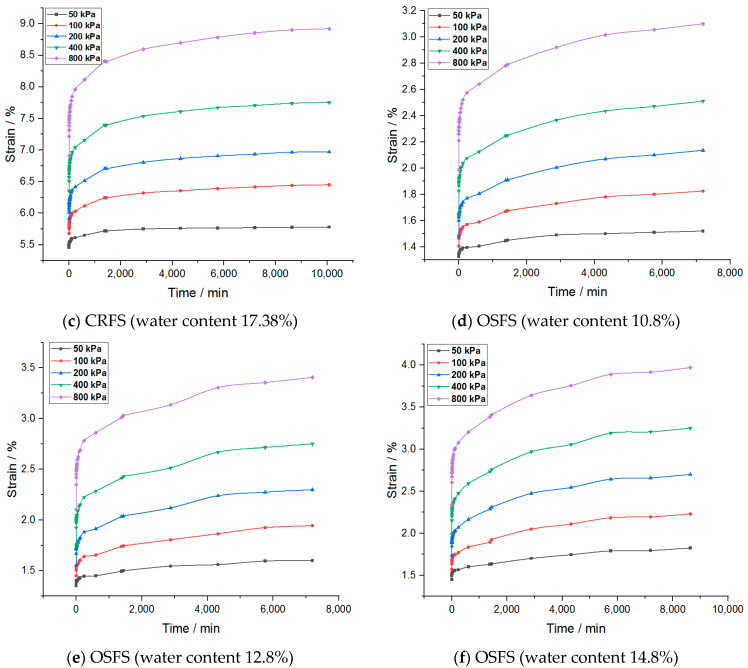
Strain–time curves of samples with different water content after process.

**Figure 6:**
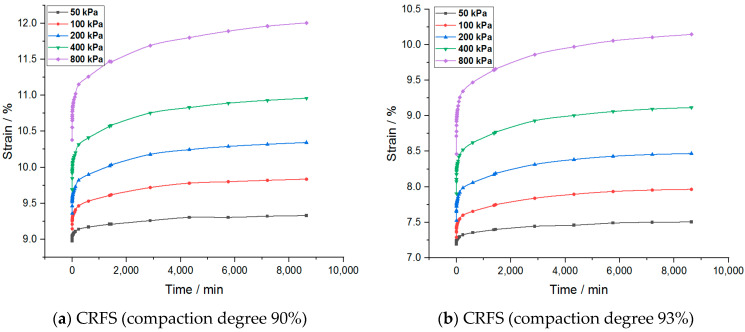

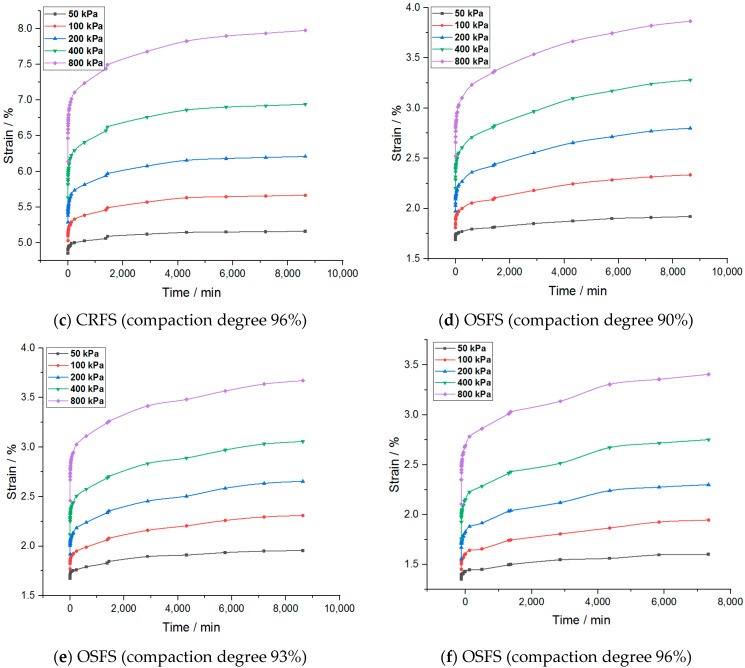
Strain–time curves of samples with different compaction degrees after processing.

Coordinates of the strain–time curve under 50 kPa were translated, and the “Chen method” was used to process the curve under subsequent loads according to the Test Methods of Soils for Highway Engineering (JTG E40-2007) [23]. The processed strain–time curve is shown in Figure 5 and Figure 6. “Chen’s method” takes the first stage load as the basis, and superimposes the creep increment of the next stage load with the same duration to obtain the creep curve with the load of 2△σ. If the cascade loading is continued, the same treatment can be performed on the basis of the upper stage. In this way, *n* creep curves at different stress levels can be obtained.

After analyzing the treated curve cluster, it can be concluded that the strain–time curve presents two stages: decay creep and constant velocity creep. Due to the restriction of the lateral limit, the soil is constantly compacted and cannot move laterally, so it cannot show the failure form of soil in the accelerated creep stage. An instantaneous strain will be generated at the moment when the load is applied, which increases with the increase of stress. When the stress level is low, the creep curve shows a stable attenuation. The amount of deformation gradually increases with the increase of time. After 4 h, the increase of deformation slows down and finally becomes stable. In the stable stage, the strain rate tends to zero with time.

**Figure 7:**
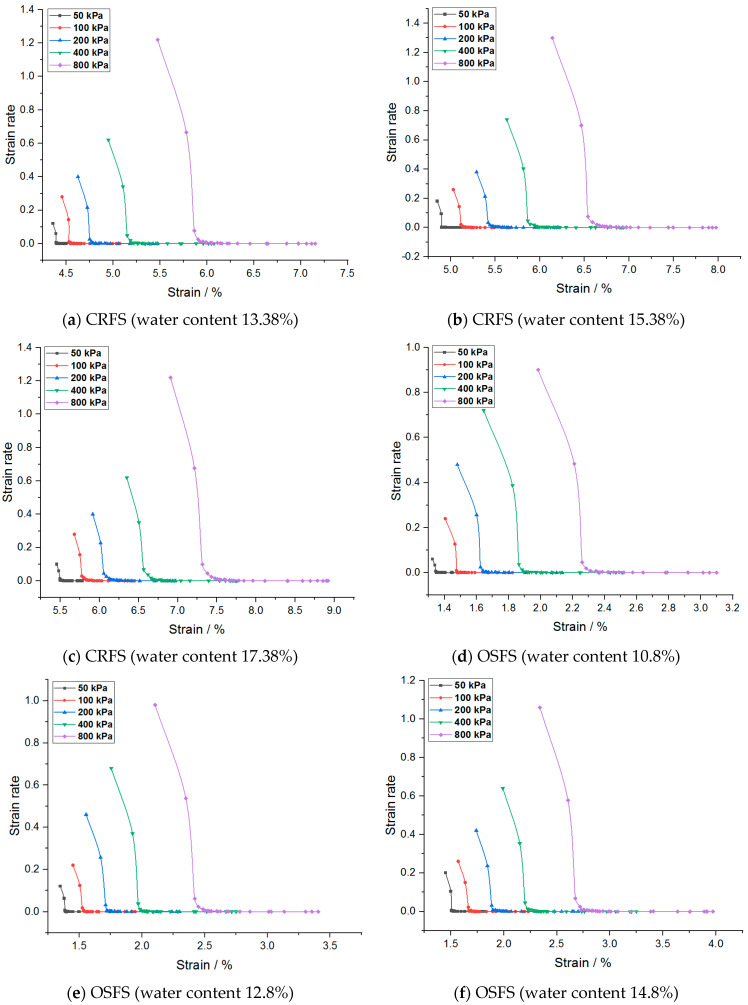
Strain rate–strain curves of modified soil with different water contents.

**Figure 8:**
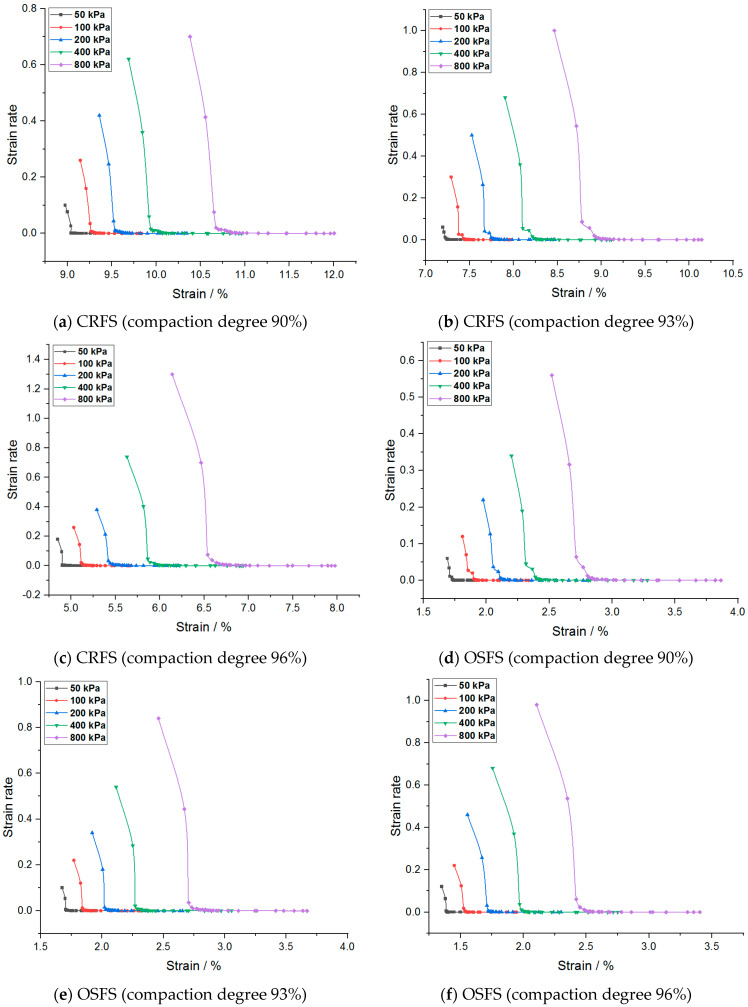
Strain rate–strain curves of modified soil with different compaction degrees.

The strain rate of the modified soil is obtained by taking the first derivative of the strain–time curve, as shown in Figure 7 and Figure 8.

It can be seen from Figure 7 and Figure 8 that the strain rate slows down gradually with the increase of strain and finally approaches zero. In addition, the initial strain rate increases with the increase of consolidation pressure, water content, and compaction degree. The strain rate of OSFS is less than that of CRFS, which is about 80% of the latter.

**Figure 9:**
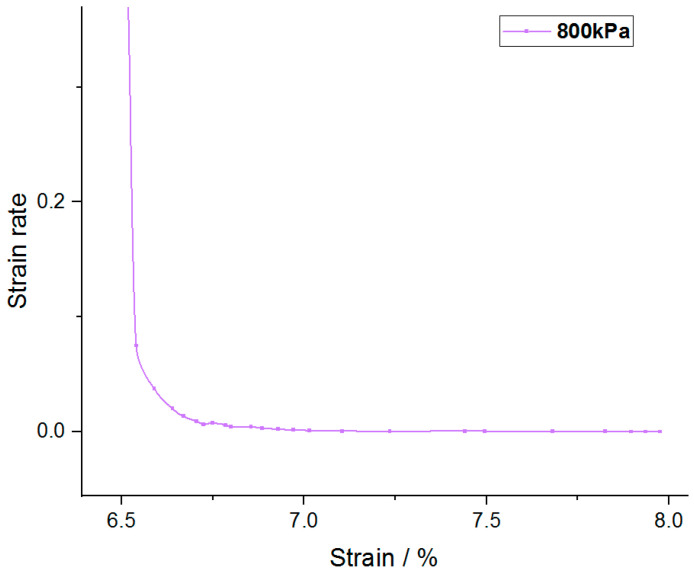
Enlarged view of end of strain rate–strain curve.

Taking Figure 7b as an example, the curve with consolidation stress of 800 kPa was enlarged as shown in Figure 9. It was found that the curve could be divided into three stages: the first stage is the period when the load is just applied and the slope of the curve is very high, which is the instantaneous deformation stage; the second stage has the inflection point, the slope decreases and the strain rate slows down gradually, which can be considered as the main consolidation stage. In the third stage, another inflection point appears in the curve, the strain rate tends to be stable and the curve gradually approaches the horizontal line, which can be considered as the secondary consolidation stage.

The curve before and after the second inflection point is approximately an oblique line, and the two extended lines intersect at a point, which can be regarded as the demarcation point of primary consolidation and secondary consolidation. According to this method, the end time of main consolidation of the two kinds of modified soil can be obtained, of which the CRFS is about 90–120 min, and the OSFS is about 240–600 min.

The secondary consolidation coefficient *C*_α_ is calculated as follows:

(1) $$C_{\alpha }=-\frac{∆e}{\log_{10}t_{2}-\log_{10}t_{1}},$$

where Δ*e* is the change of porosity ratio; *t*_2_ is the end time of the test; *t*_1_ main consolidation end time.

The secondary consolidation coefficients of the two modified soils under different water content and different consolidation pressures are shown in Table 4 and Table 5.

It can be seen that when the consolidation pressure is low, the secondary consolidation coefficient of CRFS does not change significantly with the water content, but slightly increases with the increase of water content at the higher consolidation pressure. The secondary consolidation coefficient of OSFS increases linearly with the increase of water content.

The secondary consolidation coefficient does not change significantly with the compaction degree when the consolidation pressure is low. The secondary consolidation coefficient decreases significantly with the increase of compaction degree at higher consolidation pressure. When the water content and compaction degree are constant, the secondary consolidation coefficient of the two kinds of modified soil increases in attenuation with the increase of consolidation pressure, which can be fitted by logarithmic function, and R^2^ is greater than 0.98. Among them, the secondary consolidation coefficient of OSFS is slightly larger, which is about 1.2 times that of CRFS.

### 3.2. Analysis of Pore Ratio–Stress Curve

**Figure 10:**
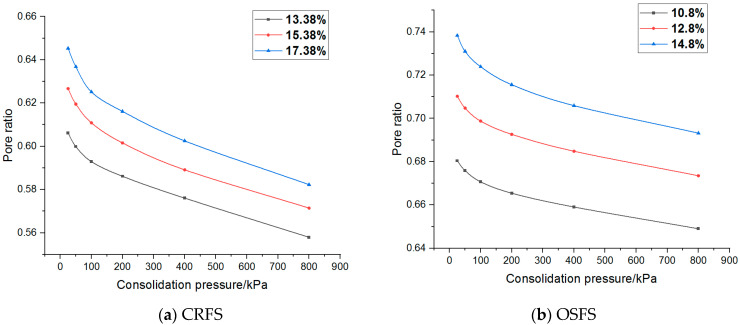
Pore ratio–stress curve of modified soil with different water contents.

**Figure 11:**
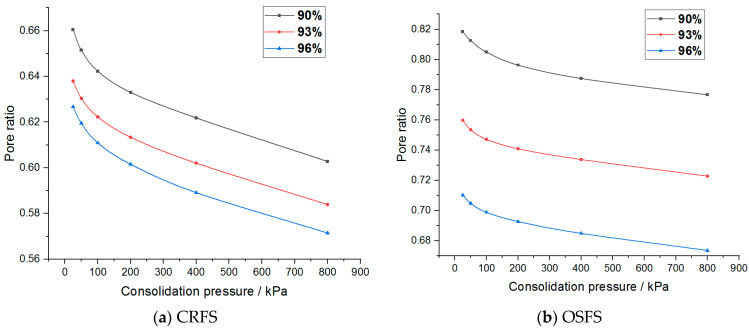
Pore ratio–stress curve of modified soil with different compaction degrees.

Pore ratio–stress curves of the two modified soils are plotted as shown in Figure 10 and Figure 11.

It can be seen from Figure 10 and Figure 11 that the relationship between the porosity ratio and the consolidation pressure of the two soils is non-linear, and the latter part of the curve is close to a straight line when the consolidation pressure is high. The curves under different conditions are parallel. The variation trend of OSFS is gentler than that of CRFS, indicating that the compressibility of CRFS is stronger.

The compression coefficient a*_v_* within a certain load range can be calculated by the pore ratio stress curve:

(2) $$a_{v}=\frac{e_{i}-e_{i+1}}{p_{i+1}-p_{i}}=\frac{\left(S_{i+1}-S_{i}\right)\left(1+e_{0}\right)/1000}{p_{i+1}-p_{i}}\;\;,$$

The compression modulus E*s* within a certain load range is calculated according to Equation (3):

(3) $$E_{s}=\frac{p_{i+1}-p_{i}}{\left(S_{i+1}-S_{i}\right)/1000}=\frac{1+e_{0}}{a_{v}}\;\;,$$

where,$\;e_{i}$ is pore ratio after compression and stabilization under a certain load; $p_{i}$ is a load value (kPa); $S_{i}$ is the settlement amount under a certain level of load (mm/m); $e_{0}$ is the pore ratio of the sample at the beginning of the test.

**Figure 12:**
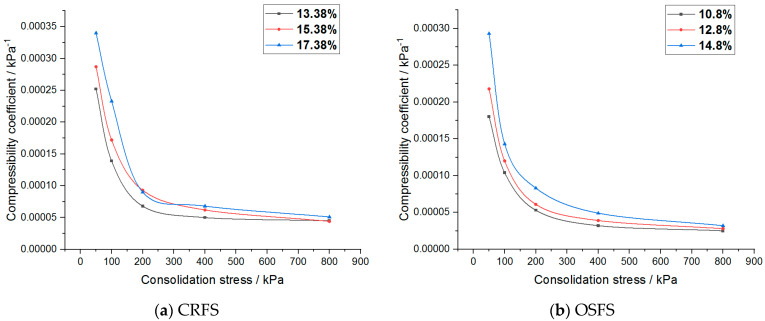
Compression coefficient of modified soil with different water contents.

**Figure 13:**
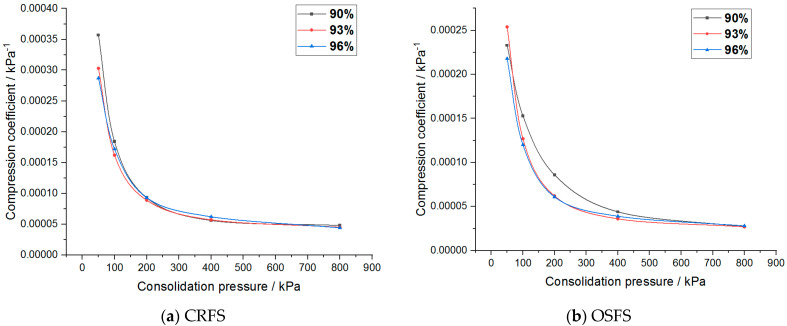
Compression coefficient of modified soil with different compaction degrees.

The compressibility coefficient of the modified soil with different water content and compactness is shown in Figure 12 and Figure 13.

It can be seen that the compression coefficient of the two modified soils increases with the increase of water content, decreases with the increase of compaction degree, decreases exponentially with the increase of consolidation stress, and finally tends to a constant value. In addition, the compressibility coefficient of CRFS is greater than that of OSFS.

### 3.3. Pore Ratio–Stress Logarithmic Curve Analysis

**Figure 14:**
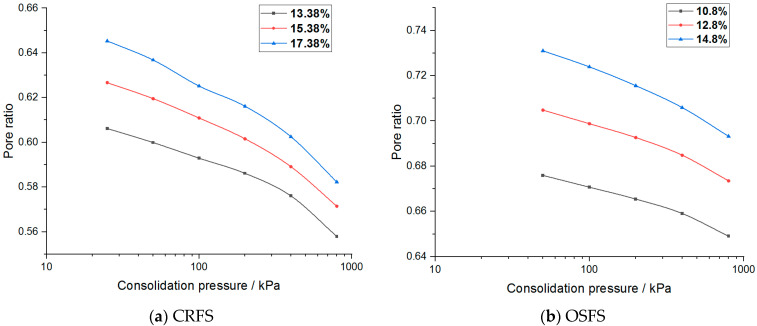
Logarithmic curve of pore ratio and stress of modified soil with different water contents.

**Figure 15:**
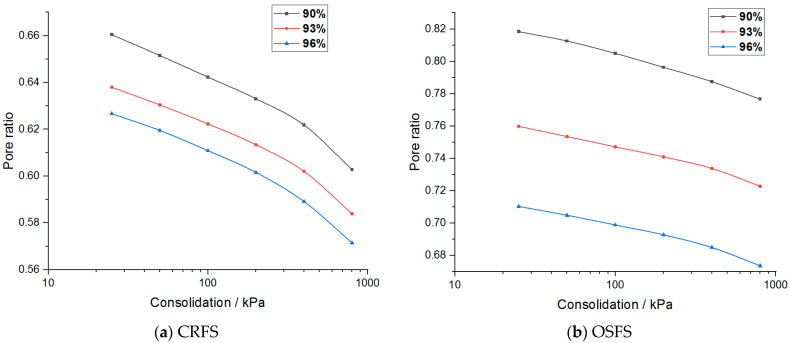
Logarithmic curve of pore ratio and stress of modified soil with different compaction degrees.

In order to obtain the consolidation yield stress of the two kinds of modified soil, the logarithmic curve of porosity ratio and stress of the modified soil was drawn, as shown in Figure 14 and Figure 15.

Consolidated yield stresses of two kinds of modified soils can be obtained by the Casagrande plotting method. The horizontal line and tangent line are respectively made at the point of the maximum curvature of the curve, and then the angle bisector of the two lines is made to extend the straight line at the end of the porose-stress logarithmic curve and intersect the angle bisector. The abscess stress value corresponding to the intersection point is the consolidated yield stress of the soil sample.

Consolidated yield stresses of the two modified soils with different water contents and compactness are shown in Table 6 and Table 7.

It can be seen from the above table that the consolidation yield stress of the two modified soils decreases with the increase of water content. The consolidated yield stress of CRFS shows little change with the increase of compaction degree, but increases with the increase of compaction degree.

The compressibility of soil is usually evaluated by the compression index C*_C_,* calculated according to the following formula:

(4) $$C_{c}=\frac{e_{i}-e_{i+1}}{\log_{10}P_{i+1}-\log_{10}P_{i}},$$

**Figure 16:**
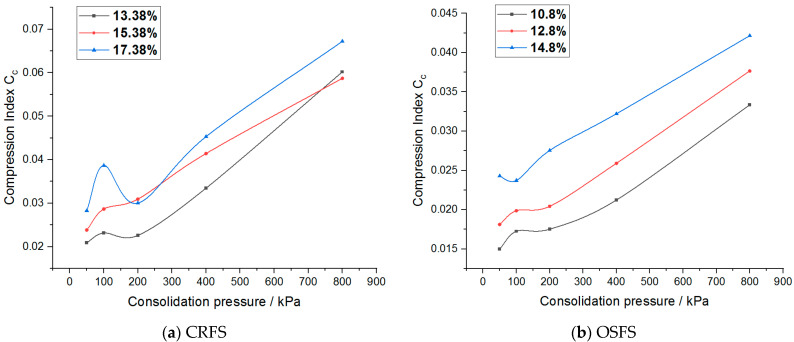
Compression index of modified soil with different water contents.

**Figure 17:**
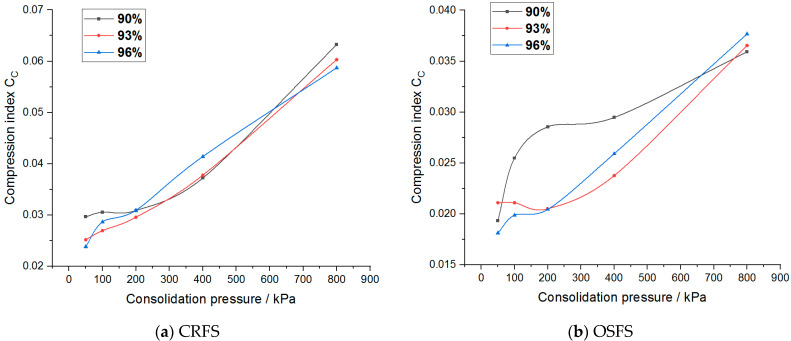
Compression index of modified soil with different compaction degrees.

The compression index of the two modified soils under different water contents and compactness is shown in Figure 16 and Figure 17.

It can be seen from the figure that the compression index of the two modified soils does not change significantly when the stress level is low, but increases linearly when the stress level is high. The compression index of the two modified soils increases with the increase of water content but has no obvious change rule with the increase of compaction degree. Moreover, the compression index of CRFS is higher than that of OSFS, but the compression index of the two modified soil is far less than 0.2, indicating that the two kinds of soil are low compressibility soils.

### 3.4. Data of Dial Indicator–Time Square Root Curve Analysis

The data of dial indicator *d* (mm) was taken as ordinate and time square root $\sqrt{t}$ was taken as abscissa and proceeded as follows. Extend the line at the beginning of the $d-\sqrt{t}\;$ curve and intersect the vertical axis at $d_{s}$ (theoretical zero).

Take another straight line through $d_{s}$, and its abscissor is 1.15 times the abscissor of the previous straight line, then the square of the time corresponding to the intersection point of the later straight line and the $d-\sqrt{t}\;$ curve is the time $t_{90}$ required for the degree of consolidation to reach 90%.

Consolidation coefficient $C_{v}$ is calculated according to Formula (5):

(5) $$C_{v}=\frac{0.848{\bar{h}}^{2}}{t_{90}}\;\;,$$

where, $C_{v}\;$ is the consolidation coefficient (cm^2^/s), calculated to three significant digits; the calculation accuracy of $\bar{h}$ is 0.01, which is equal to half of the average value of the initial and final height of the sample under a certain load.

**Figure 18:**
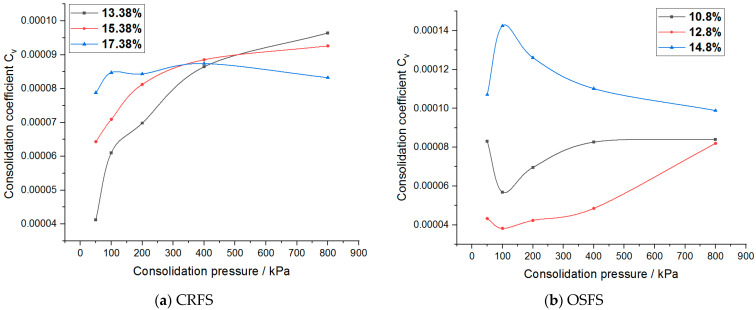
Consolidation coefficient of modified soil with different water contents.

**Figure 19:**
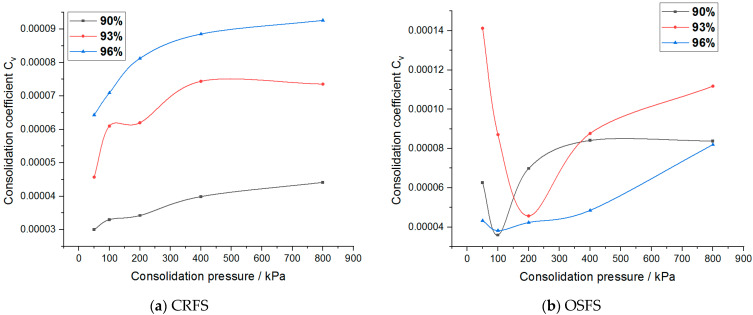
Consolidation coefficient of modified soil with different compaction degrees.

The consolidation coefficient of the modified soil with different water contents and compactness is shown in Figure 18 and Figure 19.

As can be seen from Figure 18 and Figure 19, when the consolidation pressure increases, the consolidation coefficient of CRFS shows an increasing trend. The greater the degree of compaction, the greater the consolidation coefficient. The influence of water content on the consolidation coefficient shows no obvious rule. The consolidation coefficient of OSFS with different water contents varies with the consolidation pressure but tends to a certain value. The consolidation coefficient of OSFS with different compactness decreases first and then increases with the increase of the consolidation pressure, and the effect of the consolidation pressure on the consolidation coefficient shows no obvious rule.

## 4. Conclusions

Contrastive analysis was made on the data of the consolidation-creep test of CRFS and OSFS under different water contents and compaction degrees, and the influence of water content and compaction degree on the consolidation and creep characteristics of the modified soil was evaluated. The following conclusions can be drawn:

1. The higher the water content and the lower the compaction degree, the greater the creep deformation of the modified soil. The creep deformation of OSFS is relatively small, which is about 40% of that of CRFS, and it is also less affected by water content and compaction degree;
2. The secondary consolidation coefficient of the two modified soils increases with the increase of water content, decreases with the increase of compaction degree, and increases in attenuation with the increase of consolidation pressure. The secondary consolidation coefficient of OSFS is slightly larger, which is about 1.2 times that of CRFS;
3. The compressibility coefficient of the two modified soils increases with the increase of water content, decreases with the increase of compaction degree, decreases exponentially with the increase of consolidation pressure, and finally tends to a constant value. The compressibility coefficient of OSFS is about 73% of that of CRFS;
4. The consolidation coefficient of CRFS increases with the increase of compaction degree, while the consolidation coefficient of OSFS improves with the increase of compaction degree without an obvious rule.

Water content and compaction degree are crucial to the stability of the subgrade. This paper studied the consolidation creep behavior of two kinds of modified soils under different water contents and compaction degrees. The research results provide data support and reference for practical engineering applications of the two kinds of modified soils.

## Figures and Tables

**Table 1:** Physical properties of the two modified soils.

Index	Liquid Limit (%)	Plastic Limit (%)	Plasticity Index	Optimum Moisture Content (%)	Maximum Dry Density (g/cm^3^)
CRFS	38.3	24.9	13.4	15.38	1.73
OSFS	39.4	26.9	12.5	12.8	1.67

**Table 2:** Chemical composition of the three raw materials.

Composition	SiO_2_	Al_2_O_3_	Fe_2_O_3_	CaO	MgO	Na_2_O	K_2_O	TiO_2_	Loss on Ignition
Oil Shale Residue	56.28	13.44	7.22	6.42	2.49	2.31	1.84	0.59	8.56
Fly Ash	63.55	22.00	3.09	0.92	1.08	0.75	3.08	1.10	3.19
Silty Clay	68.76	14.53	4.13	1.25	1.32	1.99	3.07	0.73	3.56

**Table 3:** Preparation scheme of modified soil specimen.

Modified Soil	Control Indicators	
	Moisture Content	Dry Density
**CRFS**	13.38%	1.6608 g/cm^3^
	15.38%	1.6608 g/cm^3^
	17.38%	1.6608 g/cm^3^
	15.38%	1.5570 g/cm^3^
	15.38%	1.6089 g/cm^3^
**OSFS**	10.80%	1.5984 g/cm^3^
	12.80%	1.5984 g/cm^3^
	14.80%	1.5984 g/cm^3^
	12.80%	1.4985 g/cm^3^
	12.80%	1.5484 g/cm^3^

**Table 4:** Secondary consolidation coefficient of modified soil with different water contents.

Consolidation Pressure	CRFS			OSFS		
	13.38%	15.38%	17.38%	10.8%	12.8%	14.8%
50 kPa	0.00186	0.00175	0.00174	0.00157	0.00246	0.00366
100 kPa	0.00382	0.00367	0.00421	0.00312	0.00468	0.00649
200 kPa	0.00504	0.00513	0.00573	0.00448	0.00638	0.00855
400 kPa	0.00609	0.0069	0.00744	0.00531	0.00772	0.01069
800 kPa	0.00823	0.00901	0.00999	0.00641	0.00898	0.01231

**Table 5:** Secondary consolidation coefficient of modified soil with different compaction degrees.

Consolidation Pressure	CRFS			OSFS		
	90%	93%	96%	90%	93%	96%
50 kPa	0.00239	0.00217	0.00175	0.00218	0.00266	0.00246
100 kPa	0.00465	0.00448	0.00367	0.00486	0.00512	0.00468
200 kPa	0.00659	0.00595	0.00513	0.00755	0.00657	0.00638
400 kPa	0.00806	0.00723	0.0069	0.00964	0.00761	0.00772
800 kPa	0.01064	0.00962	0.00901	0.01064	0.00882	0.00898

**Table 6:** Consolidation yield stress of modified soil with different water contents (kPa).

Modified Soil	CRFS			OSFS		
Water Content	13.38%	15.38%	17.38%	10.8%	12.8%	14.8%
Consolidated Yield Stresses	191	173	180	171	168	155

**Table 7:** Consolidation yield stress of modified soil with different compaction degrees (kPa).

Modified Soil	CRFS			OSFS		
Compaction Degrees	90%	93%	96%	90%	93%	96%
Consolidated Yield Stresses	179	177	173	123	149	168

## Data Availability

Not applicable.
